# LDB2 inhibits proliferation and migration in liver cancer cells by abrogating *HEY1* expression

**DOI:** 10.18632/oncotarget.21772

**Published:** 2017-10-10

**Authors:** Hongyang Yu, Ruichun Jia, Ling Zhao, Shigang Song, Jing Gu, Haogang Zhang

**Affiliations:** ^1^ Department of Radiation Oncology, The Second Affiliated Hospital, Harbin Medical University, Harbin 150086, China; ^2^ Department of Blood Transfusion, The Second Affiliated Hospital, Harbin Medical University, Harbin 150086, China; ^3^ Oncology Department of Internal Medicine, The Third Affiliated Hospital of Harbin Medical University, Harbin 150040, China; ^4^ Department of Anesthesia, The Third Affiliated Hospital of Harbin Medical University, Harbin 150040, China; ^5^ Department of General Surgery, The Second Affiliated Hospital, Harbin Medical University, Harbin 150086, China

**Keywords:** HCC, LDB2, BRD7, proliferation, HEY1

## Abstract

Hepatocellular carcinoma (HCC) was one of the most common cancers around the world, has very low 5-year survival rate. However, the mechanism of HCC occurrence and development is largely unknown. LDB2 belongs to the LIM-domain binding family and functions as an adaptor for transcriptional regulation. Here we found that LDB2 is downregulated in HCC samples. LDB2 has the ability to inhibit proliferation and migration of hepatocarcinoma cells. We found that the proliferation and migration abilities in HCC sample cells were impaired after LDB2 overexpression and vice versa. In mechanism, we found that LDB2 can recruit BRD7 to *HEY1* promoter and then block its expression. HEY1 whose expression is upregulated in HCC acts as an oncogene. In brief, our research reveals a new regulatory mechanism for hepatocarcinoma cell proliferation and migration.

## INTRODUCTION

Hepatocellular carcinoma (HCC) is one of the most common solid tumor and leads to large amounts of deaths around the world [1]. The occurrence of HCC often comes from chronic infection with hepatitis virus B or C [2]. Up to now, no effective therapy for HCC complete cure has been developed. Patients with HCC show a poor prognosis and a high recurrence rate. Quite a lot of effort has been made to explore the mechanism of HCC progression and develop effective treatments. However, the molecular mechanisms in HCC are still largely unknown.

LDB2, also known as CLIM-1, was identified as an LIM domain-associated cofactor and functions as a transcriptional regulatory factor [3-5]. LDB2 belongs to the LIM-domain binding family including LDB1 and LDB2. Though the function of LDB1, the homologous protein of LDB2, has been widely explored, the biology role of LDB2 is largely unknown. LDB1 and LDB2 bear 78% identity and 89% similarity to each other [6]. LDB1 is ubiquitously expressed while LDB2 is expressed more regionally. LIM-HDs (LIM homeodomain transcription factors) associating with LDB proteins are essential to exert biological and transcriptional activity [7, 8]. Previous study has shown that LDB2 drivers transendothelial migration of leukocytes and atherosclerosis [9]. LDB2 targets CAD (carotid artery disease) by inhibiting the activity of TEML (the transendothelial migration of leukocyte). Johnsen and colleagues reported that LDB2 has a negative effect on ERα activity and impairs ERE-dependent transcription in breast cancer [6]. Nevertheless, the roles of LDB2 in HCC remain to be elucidated. Bromodomain containing 7 (BRD7) that is a member of the bromodomain-containing protein family was acknowledged as a component of one form of the SWI/SNF chromatin remodeling complex [10, 11]. Recent studies indicated that BRD7 functioned as a chromatin remodeler and inhibited gene expression [11, 12]. And more and more evidences showed that BRD7 were downregulated in some tumor cells including epithelial ovarian carcinoma, breast cancer and colorectal carcinoma [13-15]. BRD7 was demonstrated to act as a tumor suppressor [16-18]. For example, Chen et al. demonstrated that BRD7 was a potential tumor suppressor in hepatocellular carcinoma [17]. However, the molecular mechanism of BRD7 function in HCC was still unknown. HEY1 was a classic target gene of NOTCH signaling that was shown to promote many kinds of tumor progression [19-21]. The Expression of *HEY1* can also be regulated in a NOTCH-independent way [22]. Previous study identified that HEY1 was a putative oncogene in HCC [23]. However, the function of HEY1 in HCC needs further investigation.

In this study, we showed that LDB2 inhibited proliferation and migration of hepatocarcinoma cells. Mechanistically, we found that LDB2 can promote BRD7 binding to *HEY1* promoter and then inhibits *HEY1* expression. HEY1 whose expression is upregulated in HCC functions as an oncogene. In brief, our research reveals a new regulatory mechanism for hepatocarcinoma cell proliferation and migration.

## RESULTS

### LDB2 is weakly expressed in HCC tumors

**Figure 1:**
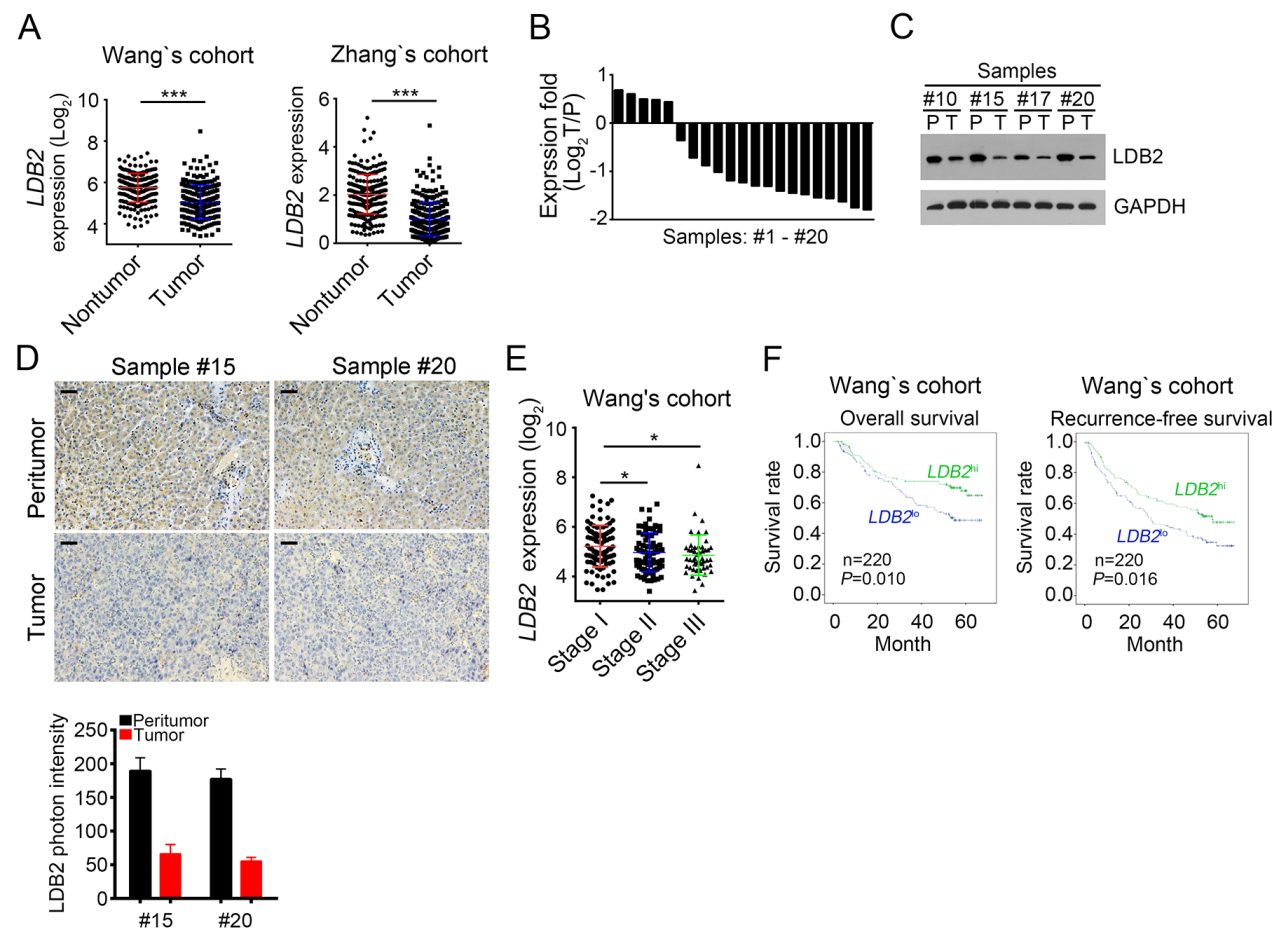
LDB2 is weakly expressed in HCC tumors **(A)** Expressionanalysis of*LDB2* in normal (n=215) and tumor tissues (n=224) according to Wang’s cohort and Zhang’s cohort. **(B)** Total RNAs were extracted from human HCC samples. Then expression levels of*LDB2* were analyzed in 20 pairs of peritumor and HCC samples by RT-qPCR. Fold changes were normalized to endogenous *Actb*. T, tumor; P, peritumor. **(C)** Expression levels of*LDB2* in peritumor and HCC samples were examined by Western blot. GAPDH was chosen for sample loading control. **(D)** Protein levels of LDB2 were analyzed by IHC in peritumor and tumor samples. Scale bars, 100μm. **(E)** Expression level of LDB2 was negatively correlated with clinical stages according to Wang’s cohort. **(F)** Expression level of LDB2 was negatively correlated with poor prognosis by Kaplan–Meier survival analysis according to Wang’s cohort. The mean expression levels of LDB2 served as cut-off values. ^*^p<0.05, ^**^p<0.01 and ^***^p<0.001 by two-tailed Student’s *t* test. All data presented are shown as means ± SD collected from three independent experiments.

To understand the mechanism that regulates the occurrence of hepatocellular carcinoma, we analyzed previous reported microarray datasets [24-27]. We found that the expression of LDB2 was downregulated in HCC samples compared with normal tissues according to the microarray data in Wang’s cohort (GSE14520) and Zhang’s cohort (GSE25097) (Figure 1A). To confirm this observation, we examined 20 pairs of HCC samples and peritumor tissues and found that LDB2 was downregulated in most HCC sample cells (Figure 1B), which was further demonstrated by western blot (WB) and immunohistochemistry (IHC) (Figure 1C and 1D). What’s more, LDB2 was expressed in HCC patients of stage III least according to Wang’s cohort (GSE14520) (Figure 1E). Importantly, HCC patients with more LDB2 expression levels had higher survival rate, and vice versa according to Wang’s cohort (GSE14520) (Figure 1F). In sum, LDB2 is downregulated in HCC samples and negatively correlated with clinical severity and poor prognosis.

### LDB2 deletion promotes liver cancer cell proliferation and migration

**Figure 2:**
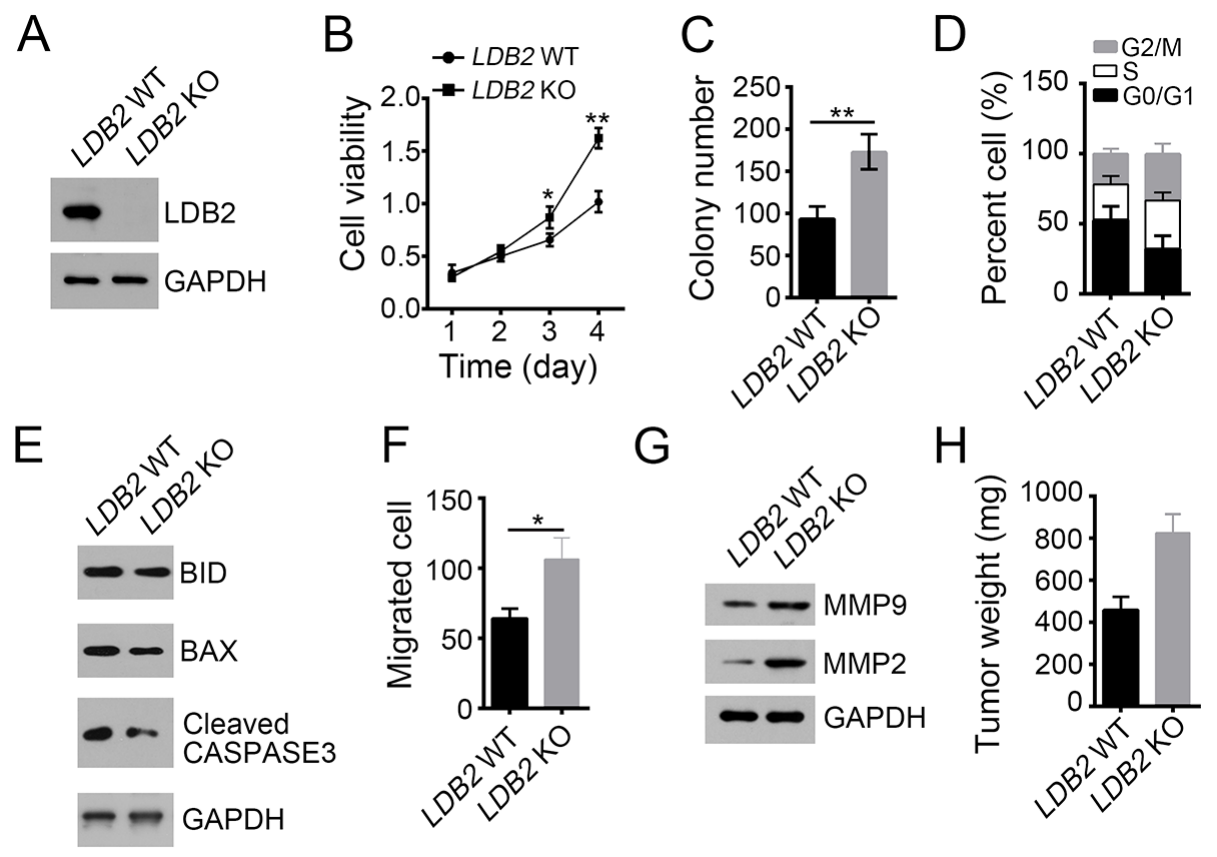
LDB2 deletion promotes liver cancer cell proliferation and migration **(A)** LDB2 was deleted in HCC samples using two individual sgRNAs through CRISPR/Cas9 technology. The deletion efficiency was validated by Western blot. **(B** and **C)** Deletion of LDB2 enhanced proliferation of HCC sample cells as examined by MTT assays and colony formation assays. **(D)** Percentages of cell-cycle distribution of WT or LDB2-deleted HCC sample cells were checked by FACS. **(E)** Percentages of cell apoptosis in LDB2-depleted HCC samples were measured by WB. **(F** and **G)** LDB2-deleted cells showed increased migration ability as measured by migration assays and WB. **(H)** The weights of tumors were measured 5 weeks post injection. 2×10^6^ WT or LDB2-deleted sample cells were injected into nude mice. ^*^p<0.05 and ^**^p<0.01 by two-tailed Student’s t test. All data presented are shown as means ± SD collected from three independent experiments.

To explore the function of LDB2 in liver cancer, we deleted it in HCC samples (Figure 2A). Through MTT assays and colony formation assays, we found that LDB2-deficient cells displayed definite increased ability of proliferation (Figure 2B and 2C). Moreover, more LDB2-deficient tumor cells entered into cell cycle (Figure 2D). Besides, LDB2 knockout led to decreased cell apoptosis (Figure 2E). To evaluate the influence of LDB2 deletion on cell migration, we performed migration assays and found that there were more migrated cells after LDB2 deletion (Figure 2F). This result was further validated by the observation of increased expression of MPP2 and MPP9 in LDB2-deleted tumor cells (Figure 2G). Consider the heterogeneity of the tumor cells, we used HCC cell line HepG2 for experiments. We found that LDB2 knockdown promoted cell proliferation in HepG2 cells (Supplementary Figure 1A). To define the function of LDB2 *in vivo*, we injected 1×10^6^ LDB2-deficient sample cells or control into BALB/c nude mice. Then tumor weights were measured 5 weeks post injection. LDB2 deletion remarkably promoted tumor formation *in vivo* (Figure 2H). Collectively, LDB2-dificiency promotes tumor proliferation *in vitro* and *in vivo*.

### LDB2 overexpression inhibits tumor cell proliferation and migration

**Figure 3:**
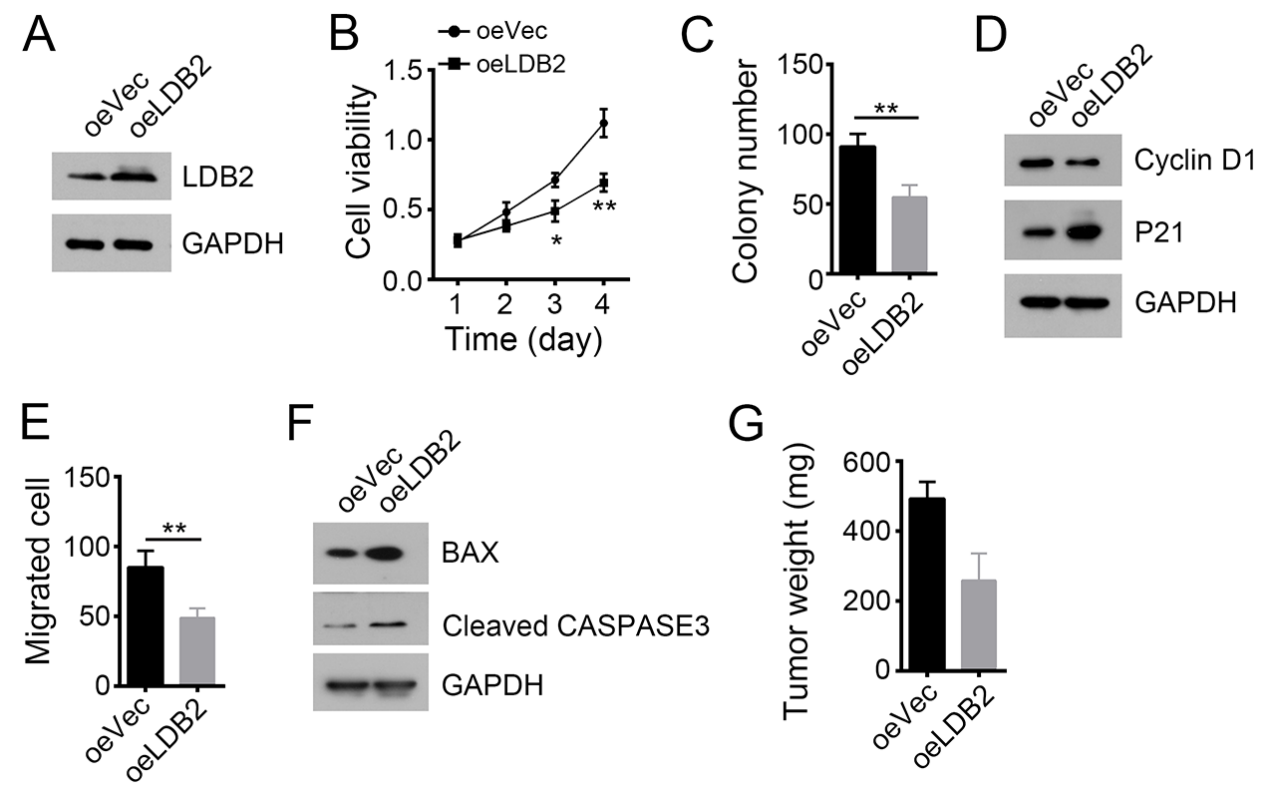
LDB2 overexpression inhibits tumor cell proliferation and migration **(A)** Overexpression of LDB2 was confirmed by WB in HCC samples. GAPDH was chosen for loading control. **(B** and **C)** Cell proliferation ability was analyzed by MTT assays and colony formation assays. **(D)** LDB2 overexpression arrested cell cycle. **(E)** Overexpression of LDB2 inhibited cell migration. **(F)** LDB2 overexpression promotes cell apoptosis as shown by WB. **(G)** LDB2 overexpression inhibited tumor formation *in vivo*. 2×10^6^ WT or LDB2-overexpressing sample cells were injected into nude mice. 5 weeks post injection, tumor weight was measured. ^*^p<0.05, ^**^p<0.01 and ^***^p<0.001 by two-tailed Student’s t test. All data presented are shown as means ± SD collected from three independent experiments.

We next overexpressed LDB2 in HCC primary tumor cells (Figure 3A). LDB2 overexpression dramatically impaired the ability of cell proliferation (Figure 3B and 3C). LDB2-overexpressing tumor cells expressed increased P21 (Figure 3D). Besides, overexpression of LDB2 significantly inhibited cell migration and promoted cell apoptosis (Figure 3E and 3F). Consequently, LDB2 overexpression remarkably reduced xenograft tumor growth (Figure 3G). Summarily, these data indicate that LDB2 plays an important role on regulating proliferation and survival of tumor cells.

### LDB2 inhibits HEY1 expression

**Figure 4:**
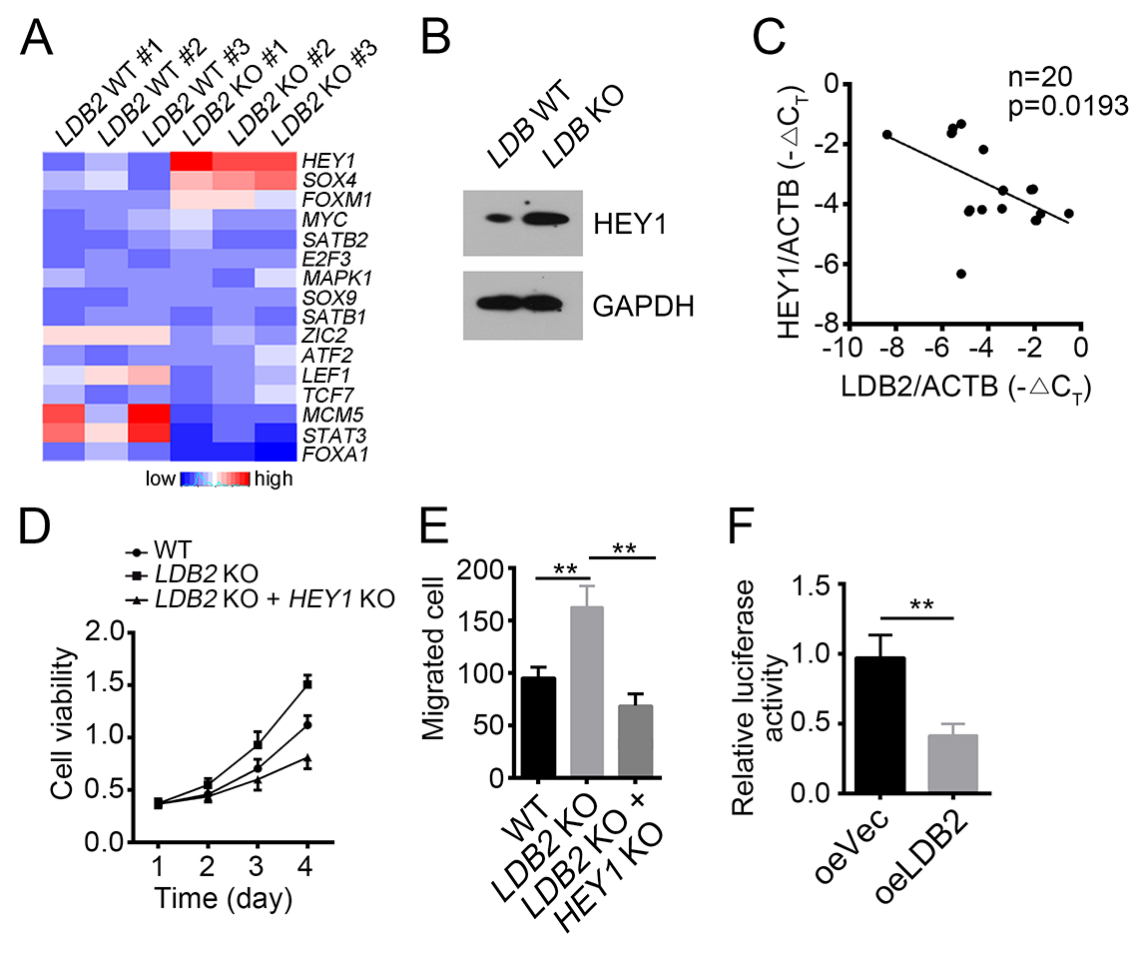
LDB2 inhibits HEY1 expression **(A)** Expression patterns of indicative transcription factors in WT and LDB-deficient HCC samples. Fold changes were normalized to endogenous *Actb*. **(B)** Protein levels of LDB2 and HEY1 were checked by WB. GAPDH was chosen for loading control. **(C)** Expression level of LDB2 was negatively correlated with HEY1 expression in HCC samples (n=20). **(D and E)** HEY1 deletion inhibited cell proliferation and migration. **(F)** LDB2 inhibited *HEY1* transcription as measured by luciferase assays. ^**^p<0.01 by two-tailed Student’s t test. All data presented are shown as means ± SD collected from three independent experiments.

Many transcription factors (TFs) have been reported to be important for HCC occurrence such as SOX4, MYC, HEY1 and ZIC2 [28-30]. To explore the molecular mechanism of LDB2 function, we checked the effect of LDB2 deletion on representative TFs by RT-qPCR. Among them, *HEY1* and *SOX4* were the most up-regulated transcription factors (Figure 4A). However, HEY1 knockdown inhibited proliferation in LDB2 KO cells while SOX4 knockdown had no effect on proliferation (Supplementary Figure 1B). We then validated that LDB2 knockout significantly enhanced HEY1 expression in HCC sample cells (Figure 4B). Besides, we analyzed the correlation between the expression levels of LDB2 and HEY1. LDB2 expression was negatively related with that of HEY1 in HCC samples (Figure 4C). Then we knocked out HEY1 in LDB2-deficient cells, and found that LDB2 deletion promoted cell proliferation but HEY1 deletion reversed it (Figure 4D). Moreover, HEY1 deletion also impaired migration ability of tumor cells (Figure 4E). To further validate that HEY1 was a target gene of LDB2, we conducted luciferase assays and found that LDB2 overexpression inhibited the luciferase activity (Figure 4F). Altogether, LDB2 inhibits tumor cell proliferation and migration by targeting HEY1.

### LDB2 recruits BRD7 to *HEY1* promoter

**Figure 5:**
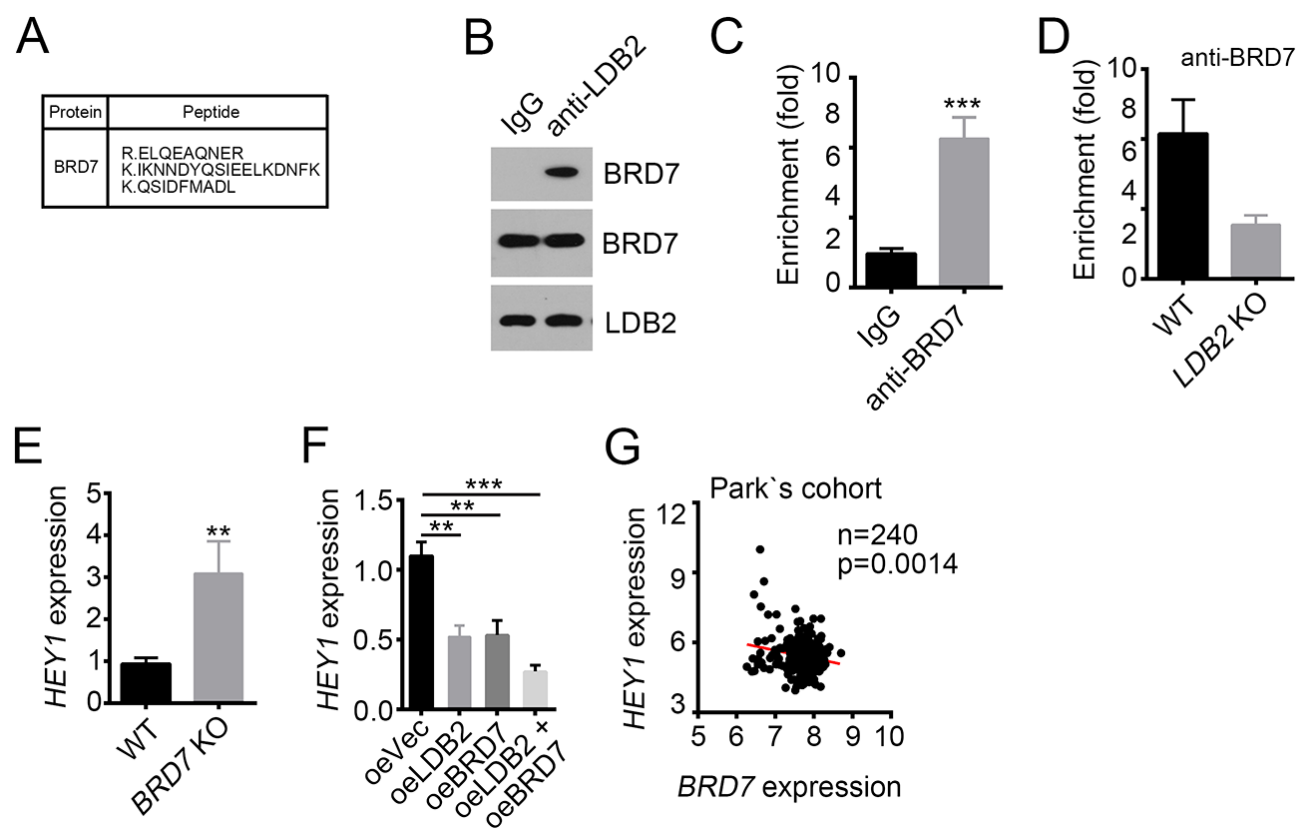
LDB2 recruits BRD7 to *HEY1* promoter **(A)** BRD7 was identified as a potential interactive protein of LDB2 by MS. **(B)** LDB2 directly associated with BRD7 as shown by co-IP assays. 1×10^7^ sample cells were lyzed and supernatant was used for incubation with antibody. **(C)** BRD7 enriched on *HEY1* promoter. **(D)** LDB2 deletion impaired the enrichment of BRD7 on *HEY1* promoter. **(E)** BRD7 deletion promoted HEY1 expression. Total RNA was extracted from WT or BRD7-deleted sample cells and RT-qPCR was conducted. Fold changes were normalized to endogenous *Actb*. **(F)** Overexpression of LDB2 or BRD7 inhibited HEY1 expression. Fold changes were normalized to endogenous *Actb*. **(G)** BRD7 expression was negatively correlated with HEY1 expression according to Park’s cohort. ^*^p<0.05, ^**^p<0.01 and ^***^p<0.001 by two-tailed Student’s t test. All data presented are shown as means ± SD collected from three independent experiments.

To further define how LDB2 regulates *HEY1* expression, we performed co-IP assays and mass spectrometry (MS). HCC sample cells were lysed and anti-LDB2 or IgG control was added. Then Protein A/G beads were used for enrichment of proteins/antibody complex, followed by SDS-PAGE and silver staining. Then the differential bands in the lane of anti-LDB2 were cut for MS. We identified BRD7 as a potential interactive protein of LDB2 (Figure 5A). Then we lysed sample cells and conducted co-IP assays to validate their interaction *in vivo* (Figure 5B). We also checked the effect of LDB2 on BRD7 expression. We found that overexpressing LDB2 had little effect on BRD7 mRNA levels in HCC (Supplementary Figure 1C). BRD7 has been reported to act as a chromatin remodeler and repress gene expression [11, 12]. To explore whether BRD7 is involved in the regulation of *HEY1* expression, we conducted ChIP assays. We found that BRD7 enriched on *HEY1* promoter (Figure 5C). Moreover, LDB2 deletion impaired the enrichment of BRD7 on *HEY1* promoter (Figure 5D). Then we deleted BRD7 in sample cells and found that BRD7-deficiency promoted *HEY1* expression (Figure 5E). On the contrary, overexpression of LDB2 or BRD7 inhibited *HEY1* expression (Figure 5F). Finally, we analyzed the expression correlation between BRD7 and HEY1 according to Park’s cohort (GSE36376), and found that their expression was negative correlated (Figure 5G). Overall, LDB2 associates with BRD7 to inhibit *HEY1* expression.

### HEY1 is correlated with clinical severity and prognosis of HCC patients

**Figure 6:**
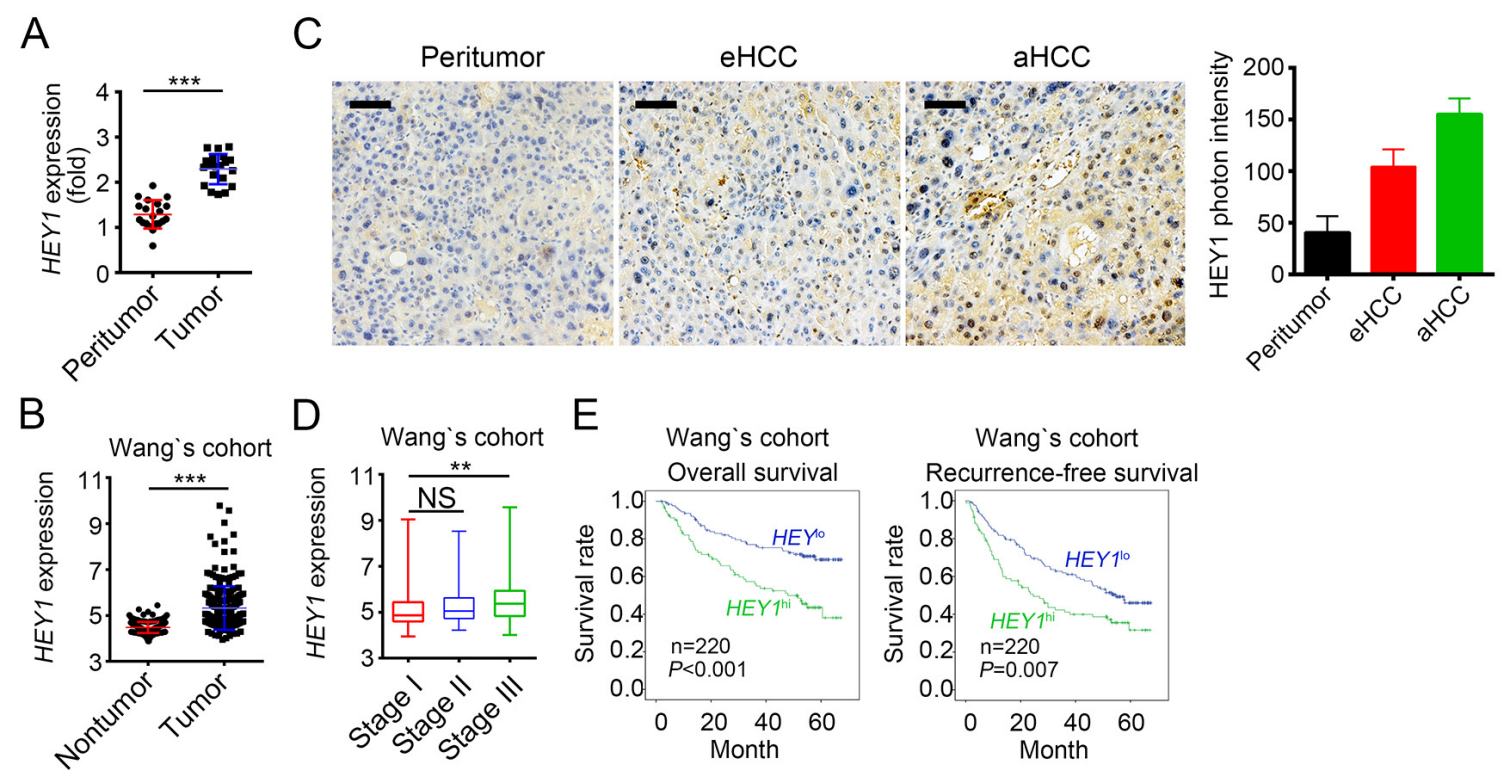
HEY1 is correlated with clinical severity and prognosis of HCC patients **(A)** HEY1 was highly expressed in HCC samples. Total RNAs were extracted from tumor and peritumor tissues. mRNA levels of *HEY1* were analyzed by RT-qPCR. Fold changes were nomalized to endogenous *Actb*. **(B)** *HEY1* was highly expressed in tumor cells according to Wang’s cohort. **(C)** The expression levels of *HEY1* were higher in eHCC (early HCC) cells and highest in aHCC (advanced HCC) cells as measured by IHC. Scale bar, 100μm. **(D)** Higher expression of *HEY1* in serious HCC samples according to Wang’s cohort. **(E)** *HEY1* expression level was positively correlated with HCC poor prognosis by Kaplan–Meier survival analyses according to Wang’s cohort. The mean expression levels of HEY1 served as cut-off values. ^**^p<0.01 and ^***^p<0.001 by two-tailed Student’s t test. All data presented are shown as means ± SD collected from three independent experiments.

To explore the physiological role of HEY1 in HCC, we analyzed the expression levels of HEY1 and found that HEY1 was highly expressed in tumor tissues (Figure 6A and 6B). HEY1 was also highly expressed in HCC tumor cells according to Wang’s cohort (GSE14520) (Figure 6C). To further define the clinical significance of HEY1 expression in HCC, we analyzed the data set of Wang’s cohort (GSE14520). We found that samples of stage III display higher expression of HEY1 (Figure 6D). To further explore the relation of HEY1 expression with HCC prognosis, we divided HCC samples into two groups according to HEY1 expression levels (mean expression level was chosen for cut-off value). Then Kaplan–Meier survival analysis was conducted and results showed that HCC patients with HEY1 higher expression had a poor prognosis and lower overall or recurrence-free survival rate, and vice versa (Figure 6E). Collectively, LDB2 and BRD7 inhibit *HEY1* expression in HCC sample cells. The expression levels of HEY1 were correlated with clinical severity and prognosis.

## DISCUSSION

HCC, the fifth most common cancer, has very low 5-year survival rate [31, 32]. Up to now, there was no good method for HCC complete treatment. So many efforts have been made to explore the molecular mechanism for hepatocarcinogenesis and the methods for HCC treatment. Nevertheless, very limited progress is achieved and the underlying mechanism of hepatocarcinogenesis is largely unknown. Therefore, it’s impending to define the mechanism underlying HCC progression. In our research, we found that LDB2 is weakly expressed in HCC sample cells. LDB2 deletion significantly promotes the proliferation and migration abilities of tumor cells. Moreover, LDB2 deficiency led to enhanced tumor propagation in mice. In mechanism, LDB2 associates with BRD7 and bound to *HEY1* promoter. Then *HEY1* transcription initiation was inhibited. Finally, high expression levels of *HEY1* are linked to clinical severity and poor prognosis.

LDB2 belongs to LIM domain binding family. As a homologous protein of LDB2, the function of LDB1 has been largely studied because of the ubiquitously expressed in tissues [6]. However, the role of LDB2 remains to be elucidated. Especially, the relationship of LDB2 with HCC is largely unknown. Through analysis of online data set of microarray about HCC samples, we found that LDB2 was remarkably downregulated in tumor cells, which suggests a tumor suppressor of LDB2. To demonstrate it, we deleted LDB2 in tumor cells. We found that LDB2-deficient cells displayed enhanced proliferation and migration ability. Besides, LDB2 knockout promoted tumor growth *in vivo*. Hence our results will shed light on the clinical correlation between LDB2 and HCC. Previous research revealed that the co-factor of LIM domains promotes breast tumorigenesis by enhancing the expression of *ErbB2* and *ErbB3* [33]. However, our results showed that LDB2 is a tumor repressor in HCC, which indicated that there may be various roles of LDB proteins in different tumors.

The LIM domain is found in a variety protein families such as the LIM-homeodomain (Lhx) and LIM-only (LMO) transcription factors [34]. Previous studies most focused on the interaction of LDB proteins with LIM domain containing proteins [35-37]. LDB proteins can also bind to other DNA-binding proteins, such as Otx family members [38]. However, how LDB2 links chromatin remodeler and gene expression is still unknown. Our study revealed that LDB2 associates with BRD7 on *HEY1* promoter and then regulate *HEY1* expression.

BRD7 that is a subunit of polybromo-associated BRG1-associated factor (PBAF)- specific SWI/SNF chromatin remodeling complexes contains an evolutionally conserved bromodomain [11]. This domain containing proteins can regulate gene transcription [39]. [4]. BRD7 has been demonstrated to be important for the transcriptional activation or repression of target genes such as P53 [40, 41]. Accumulating evidence showed that BRD7 is involved in multiple cancers and serves as a tumor suppressor, including HCC [17]. However, the fashion of BRD7 function in HCC remains to be explored. We showed that BRD7 binds to HEY1 promoter and represses its transcription. HEY1 was a classical target gene of NOTCH signaling. It can also be regulated in a NOTCH-independent manner [22]. NOTCH signaling is involved in various cancers and has been extensively explored. Nevertheless, the function of HEY1 seems to be ignored. We showed that the expression of *HEY1* was repressed by LDB2 and BRD7. Higher expression of *HEY1* means poorer prognosis. Our study showed that overexpressing LDB2 and BRD7 in the meantime seems to further reduce the expression of HEY1. So other proteins may also interact with BRD7 to regulate HEY1 expression and function in HCC, which needs to be investigated further.

Summarily, our research demonstrates that LDB2 can inhibit tumor cell proliferation and migration by downregulating *HEY1* expression. Therefore, this finding gives rise to a new insight into the mechanism of hepatocarcinogenesis.

## MATERIALS AND METHODS

### Patient samples

20 pairs of Peritumor and HCC tissues involved in this study were obtained by laser microdissection from the Third Affiliated Hospital of Harbin Medical University. For cell culture, sample tissues were cut into small pieces and then digested with collagenase IV for 60min at 37 °C. Consents approving the use of the tissues for this research were obtained from all patients. All the experiments were approved by the Third Affiliated Hospital of Harbin Medical University. The study protocol was approved by the Third Affiliated Hospital of Harbin Medical University.

### Cell lines and cell culture

293T cells from ATCC and HCC sample cells were cultured in DMEM medium containing 10% fetal bovine serum (FBS; Invitrogen), 100 μg/ml penicillin and 100U/ml streptomycin.

### CRISPR/Cas9 mediated knockout

We used CRISPR/Cas9 approach for LDB2, BRD7 and HEY1 deletion. sgRNA was designed through online CRISPR Design Tool (http://tools.genome-engineering.org). Then we cloned sgRNAs into LentiCRISPRv2 plasmid (52961) purchased from Addgene. For lentivirus production, 293T cells were transfected with LentiCRISPRv2, pMD2.G (Addgene, 12259) and psPAX2 (Addgene, 12260). A pair of guide RNAs was used simultaneously for LDB, BRD7 or HEY1 deletion. The HCC cells infected with lentivirus were treated with puromycin and then monoclonalization was conducted. sgRNA sequences are as follows: LDB2 #1: 5'-GGCACACGTCCTCAATGCTC-3'; LDB2 #2:5'-GTGCCACACGCTGTAATTTG-3'; BRD7 #1: 5'-AAGCGCGTCGATTAAAATCG-3'; BRD7 #2: 5'-GGATTACAAGTGTGGGCTAC-3'; HEY1 #1: 5'-GTTATCATCGCGGAGCTTTT-3'; HEY1 #2: 5'-GAGTCCCGAGCCCCTACATT-3'.

### Antibodies

Anti-LDB2 (sc-101088), anti-BRD7 (sc-376180) and anti-HEY1 (sc-134362) were purchased from Santa Cruz Biotechnology.

### Apoptosis analysis

Apoptosis analysis was conducted through Annexin V-FITC/PI apoptosis detection kit (eBiosciences) by FACS analysis.

### Xenograft tumor formation

We purchased six weeks old female BALB/c nude mice from HFK Biosciences and maintained under pathogen-free conditions with approval by the Institutional Committee of the Third Affiliated Hospital of Harbin Medical University. For tumor propagation analysis, 2×10^6^ *LDB2*-deficient cells were subcutaneously injected into mice. Tumor weight was analyzed on week 5 after injection. Animal experiments were performed in accordance with relevant guidelines and regulations of the Institutional Animal Care and Use Committees at the Third Affiliated Hospital of Harbin Medical University, and protocols were approved by the Institutional Animal Care and Use Committees at the Third Affiliated Hospital of Harbin Medical University.

### MTT assay

In MTT assays, 1×10^3^ cells were seeded into 96-well plates. Cell proliferation was measured by MTT assays at different time points. MTT (20 μl, 5 mg/ml) (Sigma, USA) was added into each well and incubated for 4 h at 37°C. Then 150 μl DMSO was added to solubilize the crystals. To determine cell viability, the absorbance (540 nm) was measured.

### Colony formation and migration assay

For colony formation assays, 2×10^3^ cells were seeded into a 6-well plate and incubated for 12 days at 37°C. And then the cells were fixed in 90% ethanol and stained with crystal violet solution. The formed colonies were counted.

For cell Migration assays, the transwell filter chambers (Costar, Corning, NY) were used according to the manufacturers’ instructions. Briefly, 2×10^5^ cells in serum-free medium were added into the top chamber. the lower chamber containing medium supplemented with 10% FBS. After cultured for 12 h, cells on the lower surface were stained and analyzed in six random fields for each group.

### Real-time quantitative PCR

Total RNAs were extracted with TRIzol according to the manufacturer’s protocol. Then cDNA was synthesized with the M-MLV reverse transcriptase (Promega). Then mRNA transcripts were analyzed with ABI 7300 qPCR system using specific primer pairs. Relative expressions were calculated and normalized to endogenous *Actb*. Primers used were available if requested.

### Chromatin immunoprecipitation (ChIP) assay

Cells were cross-linked with 1% formaldehyde at 37 °C for 10 min, washed twice with PBS, lysed with SDS lysis buffer and sonicated into 300–500 bp DNA fragments. Lysates were incubated with 4 μg antibody overnight at 4 °C. Then Protein A Agarose/Salmon Sperm DNA (50% Slurry) beads were added for another 4 h. After washed, DNA was eluted from beads and purified. Enriched DNA was checked by q-PCR. Primers used for *HEY1* promoter analysis were described before [42] and as follows: forward: 5'-TCAGTGTGTGCGGAACGCAAG-3'; reward: 5'- TTCTTCACCTCGATGGTCTCGTC-3'.

### Luciferase reporter gene assays

The Dual-Luciferase Reporter Assay System (Promega) was used for luciferase reporter assay. Cells were cultured on 24-well plates at 3×10^4^ cells per well. The indicated regions of *HEY1* promoter (-2000∼0 bp: upstream of TSS region) were cloned into pGL3 plasmid. 1 ng pRL-TK was co-transfected into cells. After 24 hours, cells were lysed and detected according to manufacturer’s instructions.

### Cohort analysis

Online-available data sets were available from NCBI (https://www.ncbi.nlm.nih.gov/gds/). Three online available cohorts used were as follows: Wang’s cohort (GSE14520) and Zhang’s cohort (GSE25097).

### IHC staining

The method has been reported before [43]. In brief, HCC sections were deparaffinized, rehydrated and blocked with 3% H_2_O_2_ for 15 min and antigen retrieval with Tris-EDTA buffer (10 mM, pH 8.0) at 121°C for 5min. After 30 min incubation in 5% goat serum, the sections were incubated in primary antibodies overnight, washed with PBS and then incubated in HRP-conjugated secondary antibodies. The final detection was conducted with the substrate detection of HRP. Finally the sections were stained using haematoxylin.

### Co-immunoprecipitation and mass spectrometry

HCC sample cells were lysed with RIPA lysis buffer and anti-LDB2 or IgG control was added for incubation at 4 °C overnight. Then ProteinA/G beads were added and incubated at 4 °C for 2h. Then beads were collected and IP components were separated by SDS-PAGE, followed by silver staining. Differential bands enriched by anti-LDB2 were collected for mass spectrometry (LTQ Orbitrap XL).

### Statistical analysis

All statistical analyses were performed using the Statistical Package for the Social Sciences version 20.0 software (SPSS Inc., Chicago, IL, USA). The results are shown as the mean ± SD and were analyzed using Student’s t-test. All tests were two-sided, and P < 0.05 was considered statistically significant.

## SUPPLEMENTARY MATERIALS FIGURE


