# Supplementary material for: LDB2 inhibits proliferation and migration in liver cancer cells by abrogating HEY1 expression
Source: Oncotarget. 2017 Oct 10;8(55):94440–9. doi: 10.18632/oncotarget.21772 (PMC5706886; doi:10.18632/oncotarget.21772)
Supplement: Supplementary file 1 [file oncotarget-08-94440-s001.pdf]

LDB2 inhibits proliferation and migration in liver cancer cells by abrogating *HEY1* expression

SUPPLEMENTARY MATERIALS

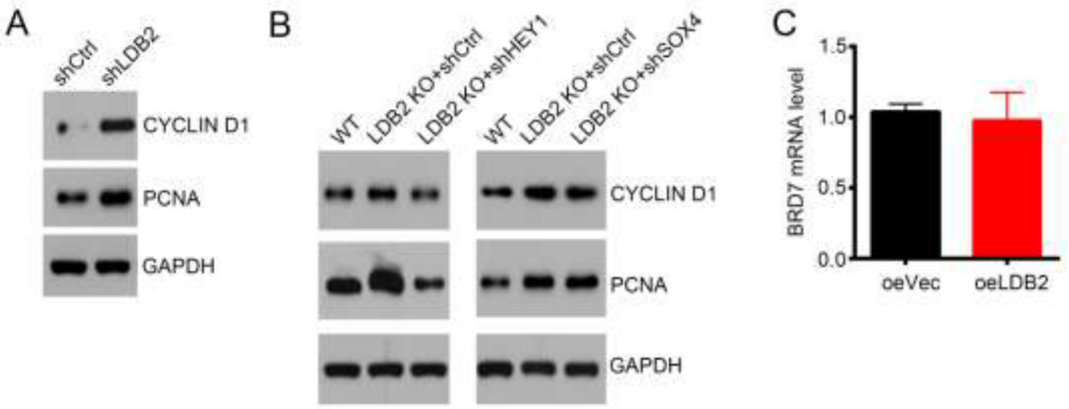

Supplementary Figure 1: LDB2 inhibited cell proliferation in HCC. **(A)** LDB2 knockdown promoted cell proliferation in HepG2 cells. shCtrl or shLDB2 HepG2 cells were lyzed and the protein levels of CYCLIN D1 and PCNA were checked by Western blot. GAPDH was loading control. **(B)** HEY1 knockdown inhibited cell proliferation in LDB2 KO sample cells. **(C)** Overexpressing LDB2 had no effect on BRD7 mRNA level. All data presented are shown as means ± SD collected from three independent experiments.
